# Supplementary material for: Synaptic basis of a sub-second representation of time in a neural circuit model
Source: Nat Commun. 2022 Dec 22;13:7902. doi: 10.1038/s41467-022-35395-y (PMC9780315; doi:10.1038/s41467-022-35395-y)
Supplement: Supplementary file 1 — Supplementary Information [file 41467_2022_35395_MOESM1_ESM.pdf]

Supplementary Figures

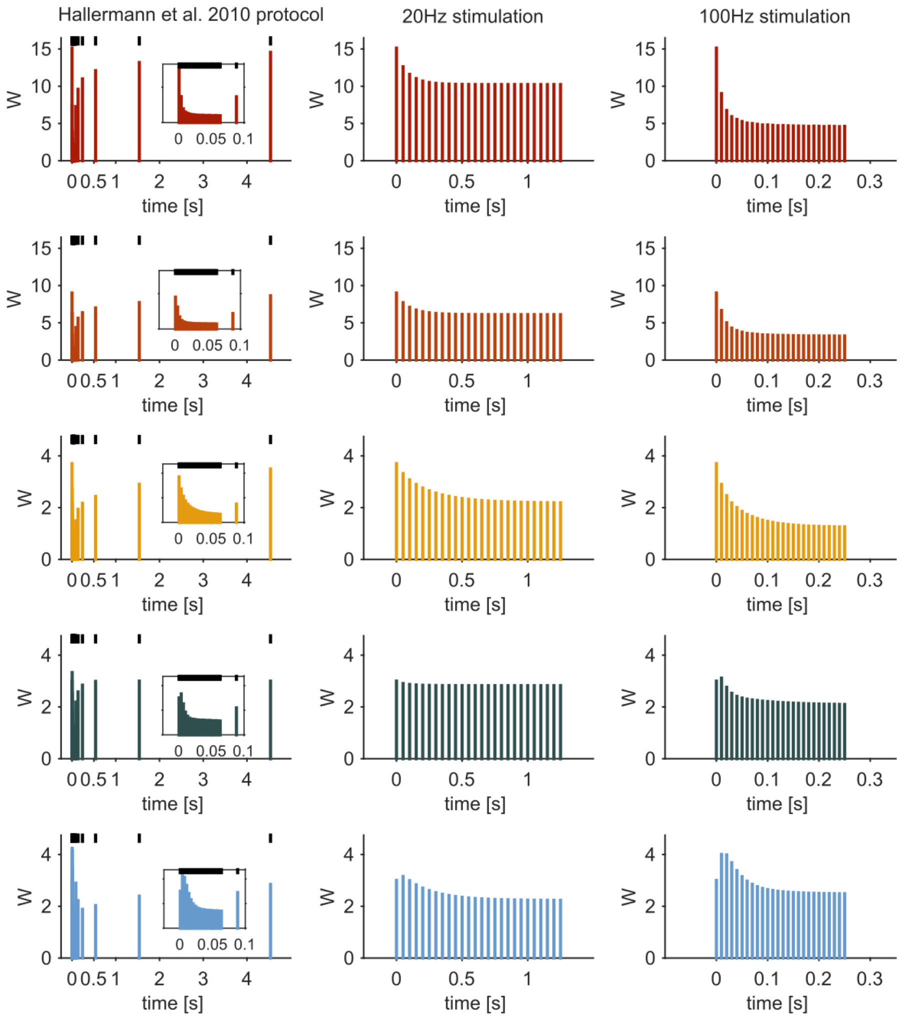

Supplementary Figure 1 *Behavior of model synapses subjected to different stimulation protocols. Each row represents one of the five synapse types from* ***Fig. 1****. First column: average synaptic weight in response to 300 Hz train followed by increasing intervals ranging from 25 ms to 5 s as in ref.*[38]*. Inset: zoom on 300 Hz train. Second and third column: average synaptic weight in response to trains of 26 stimuli at 20 Hz and 100 Hz, respectively, similar to ref.*[39]

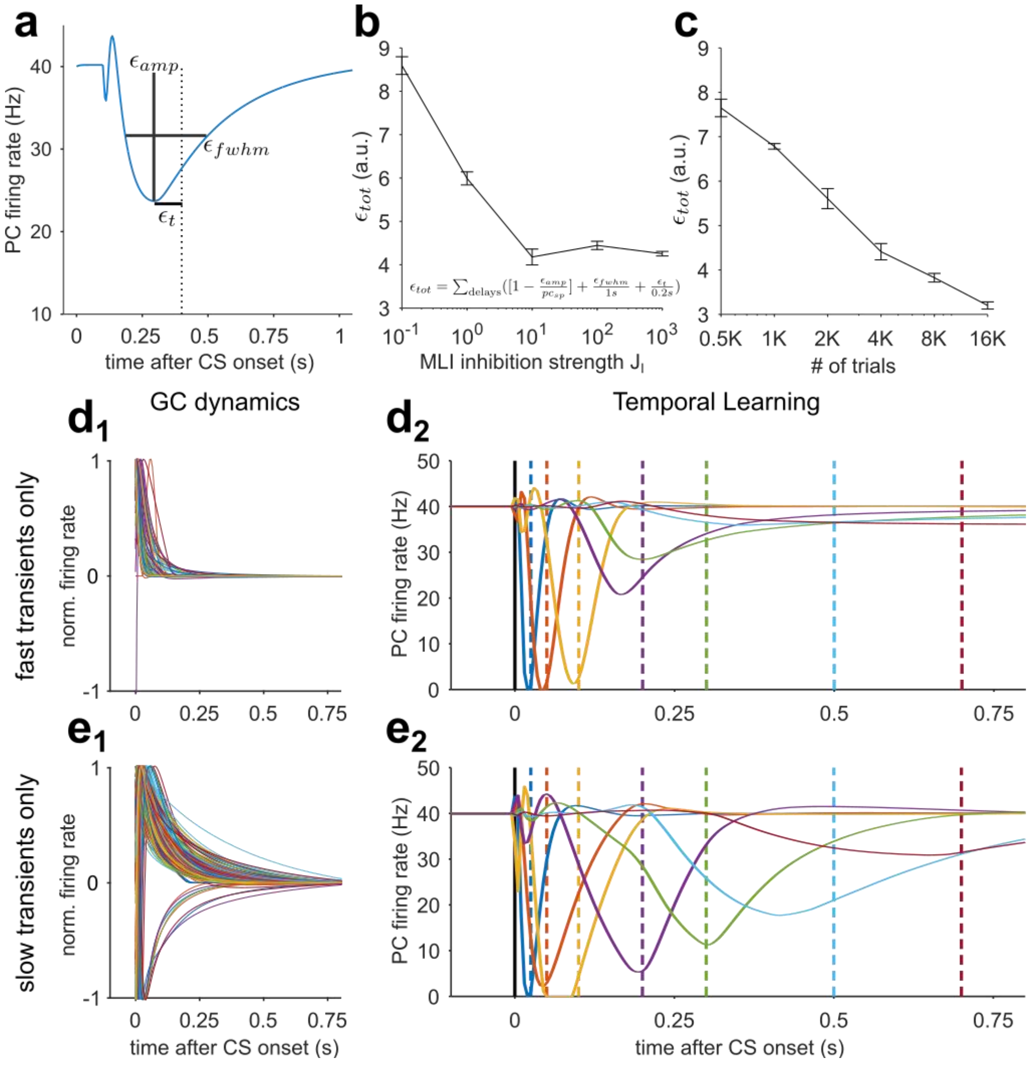

***Supplementary Figure 2 (a)** Definition of error components used to assess learning performance. **(b)** Learning error as a function of MLI-PC inhibition strength. The total error is computed from the individual components according to the formula shown. Black line represents the average over 20 realizations of the network in **Fig.2**. Error bars are SEM. **(c)** Same as **b** for learning error as a function of number of learning iterations. **(d_1)** As in **Fig.2e**, but without GCs whose transients decayed to 10% of their peak values in more than 150 ms. **(d_2)** Learning performance when using the temporal basis from panel **d_1**. **(e_1)** As in **Fig.2e**, but without GCs whose transients decayed to 10% of their peak values in less than 150 ms. **(e_2)** Learning performance when using the temporal basis from panel **e_1**.*
Note that learning of short delays was not completely abolished because our manipulations did not affect the rising phases of the GC transients.

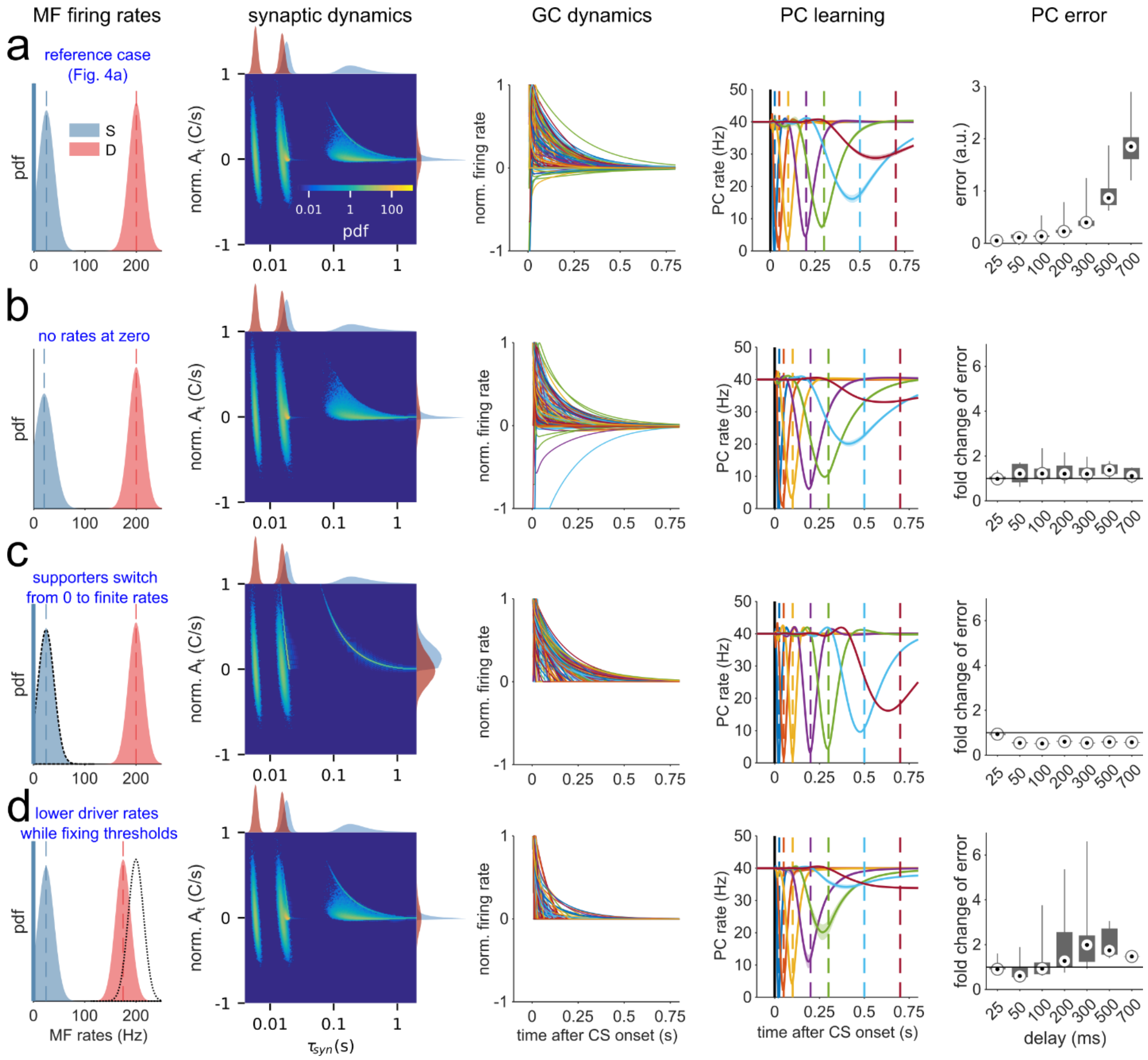

Supplementary Figure 3 *Additional examples of MF rate distributions and their impact on learning.* ***(a)*** *Same as* ***Fig. 4a*** *(i.e. reference case, see legend of* ***Fig. 4*** *for details).* ***(b)*** *Without zero firing rates.* ***(c)*** *Simulations in which all supporter inputs are set to zero before the CS and switch to finite firing rates during the CS.* ***(d)*** *Simulations in which GC thresholds are set as in* ***a*** *(blue and dashed firing rate distributions), but eyelid conditioning is carried out with lower driver inputs (blue and red firing rate distributions).*

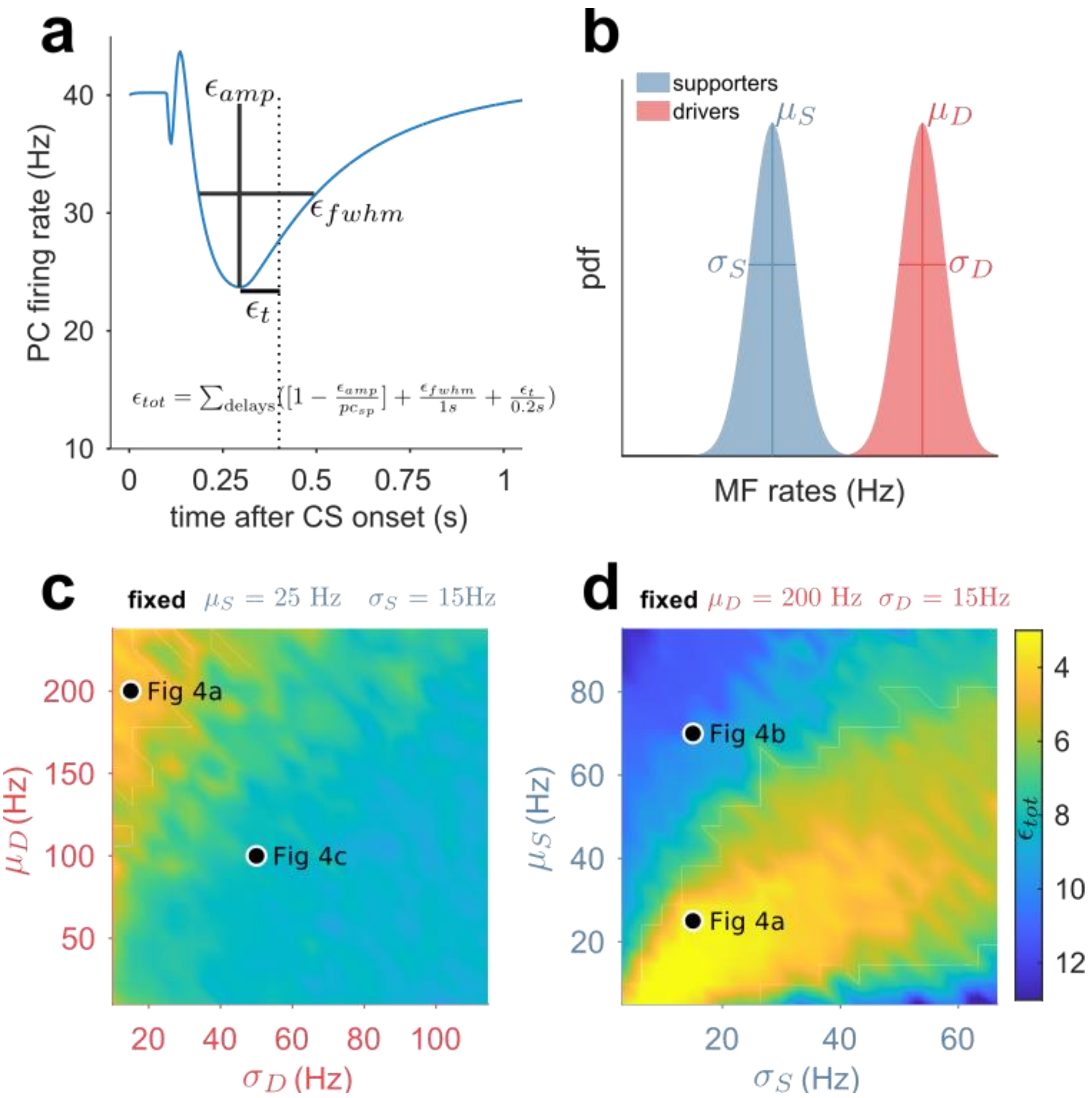

Supplementary Figure 4 *Scan of MF firing rate parameters show distinct roles for driver and supporter inputs.* ***(a)*** *Definition of error components used to assess learning performance. The total error is computed from the individual components according to the formula shown.* ***(b)*** *Definition of the MF firing rate parameters.* μ_D, μ_S *and* σ_D, σ_S *denote means and standard deviations of the MF firing rate distributions of drivers (red) and supporters (blue) respectively.* ***(c)*** *Scan over driver parameters while keeping supporter parameters fixed.* ***(d)*** *Scan over supporter parameters while keeping driver parameters fixed. In* ***c*** *and* ***d*** *the total learning error is color coded and parameter configurations corresponding to rows in* ***Fig. 4*** *are indicated by black dots.*

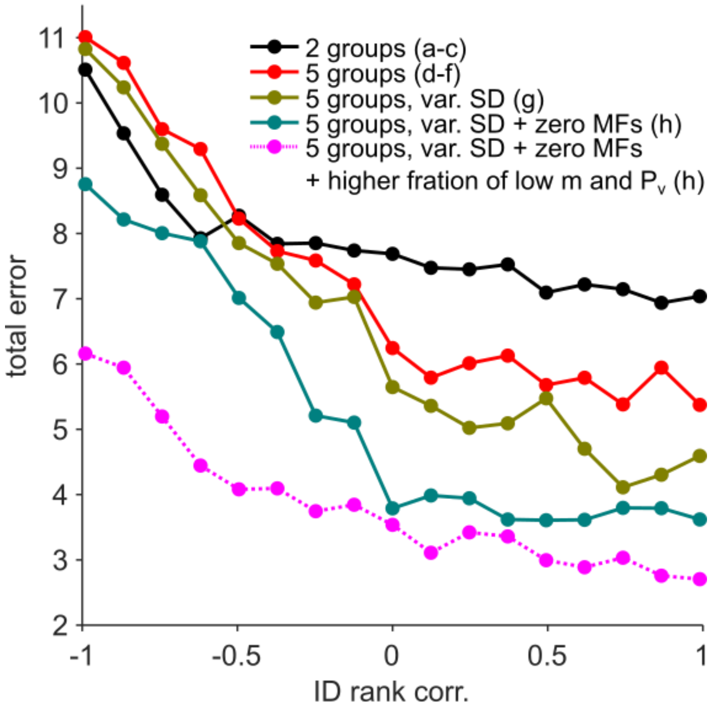

Supplementary Figure 5 *Average total error over seven delay intervals for varying rank-correlation between the m category and the p_v category for all scenarios shown in* ***Fig. 5****.*

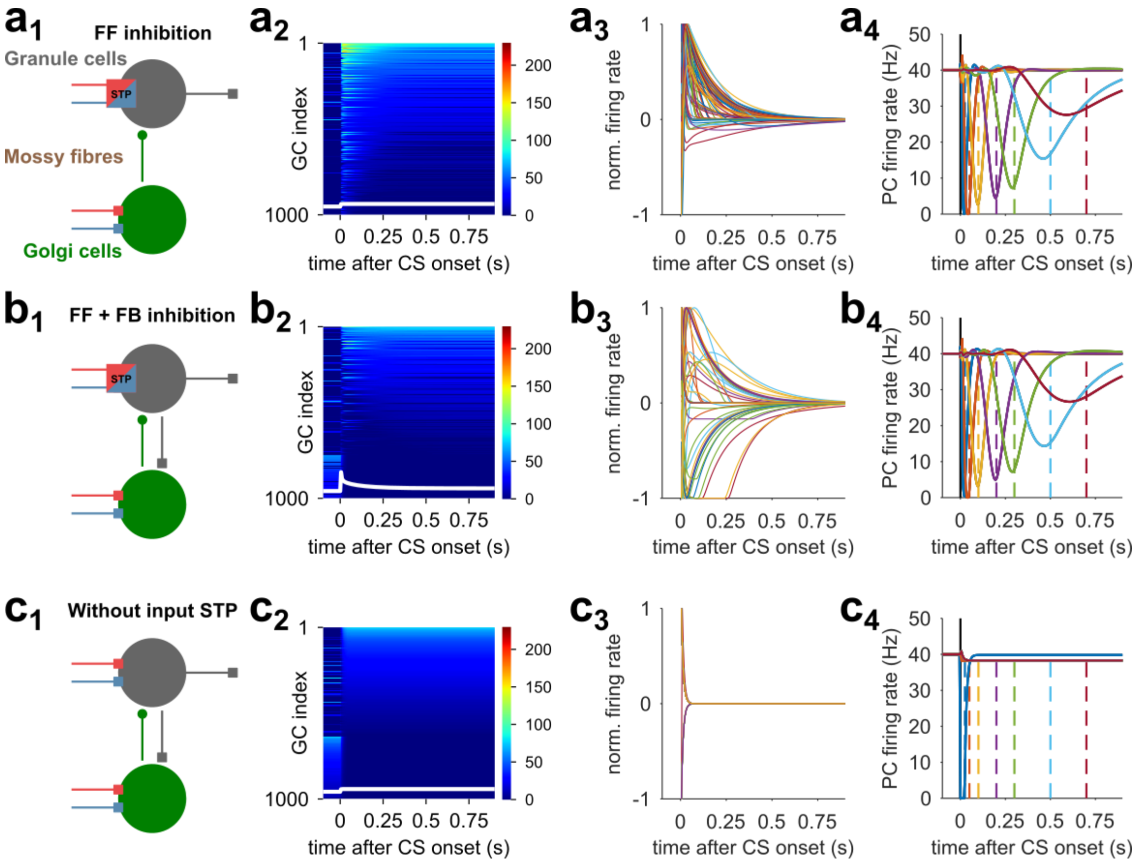

Supplementary Figure 6 *Eylid learning is not significantly affected by simple GoC feedback.* ***(*a1*)*** *Scheme of CC input layer with GoC inhibition that acts in a purely feed-forward manner. This configuration is functionally identical to the reduced model used in the main text.* ***(*a2, a3*)*** *GC temporal basis (as in* ***Fig. 2c****). White line in* **a2** *indicates GoC activity.* ***(*a4*)*** *PC eyelid response learning.* ***(*b1-4*)*** *Same as row* ***a****, but with GC-GoC feedback connections.* ***(*c1-4*)*** *Same as row* **b***, but without MF-GC STP.*

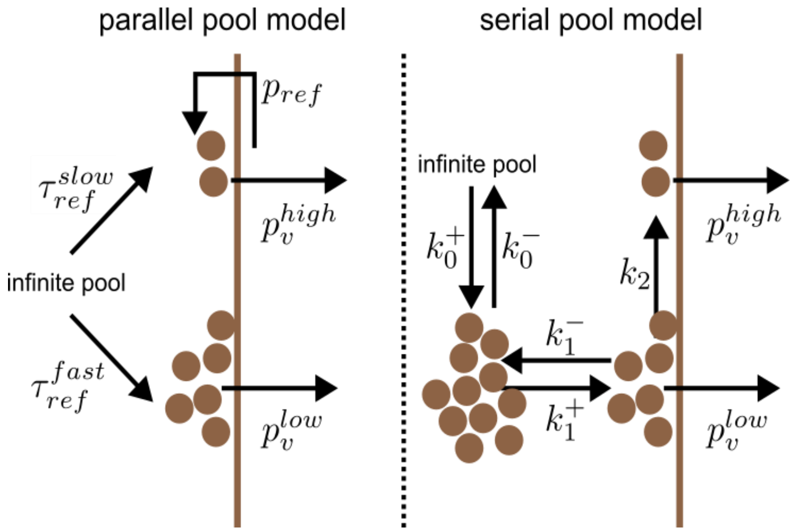

Supplementary Figure 7 *Scheme comparing the parallel pool model used here with the serial pool model from ref.*[38]*. The symbols* k_0^+, k_0^-, k_1^+, k_1^- *and* k_2 *are rate constants described in ref.*[38].
